# Refining the *Ciona intestinalis* Model of Central Nervous System Regeneration

**DOI:** 10.1371/journal.pone.0004458

**Published:** 2009-02-12

**Authors:** Carl Dahlberg, Hélène Auger, Sam Dupont, Yasunori Sasakura, Mike Thorndyke, Jean-Stéphane Joly

**Affiliations:** 1 Department of Marine Ecology, Göteborg University, Fiskebäckskil, Sweden; 2 U1126/INRA 〈〈Morphogenèse du système nerveux des chordés〉〉 group, DEPSN, UPR2197, Institut Fessard, CNRS, Gif sur Yvette, France; 3 Shimoda Marine Research Center, University of Tsukuba, Shimoda, Shizuoka, Japan; Centre de Regulacio Genomica, Spain

## Abstract

**Background:**

New, practical models of central nervous system regeneration are required and should provide molecular tools and resources. We focus here on the tunicate *Ciona intestinalis*, which has the capacity to regenerate nerves and a complete adult central nervous system, a capacity unusual in the chordate phylum. We investigated the timing and sequence of events during nervous system regeneration in this organism.

**Methodology/Principal Findings:**

We developed techniques for reproducible ablations and for imaging live cellular events in tissue explants. Based on live observations of more than 100 regenerating animals, we subdivided the regeneration process into four stages. Regeneration was functional, as shown by the sequential recovery of reflexes that established new criteria for defining regeneration rates. We used transgenic animals and labeled nucleotide analogs to describe in detail the early cellular events at the tip of the regenerating nerves and the first appearance of the new adult ganglion anlage.

**Conclusions/Significance:**

The rate of regeneration was found to be negatively correlated with adult size. New neural structures were derived from the anterior and posterior nerve endings. A blastemal structure was implicated in the formation of new neural cells. This work demonstrates that *Ciona intestinalis* is as a useful system for studies on regeneration of the brain, brain-associated organs and nerves.

## Introduction

Few model systems are currently available for studies of central nervous system (CNS) regeneration. Retina regeneration and partial brain ablations in teleostean fish are two situations in which defined parts of the central nervous system reform after removal [1], [2]. The Teleostean CNS also maintains numerous neurogenic zones in adulthood that generate new neurons throughout life [3]–[5]. Urodeles and frogs can regenerate the lens and spinal cord at tadpole stages. However, this capacity is lost in adults, and this loss is correlated with a decrease in neurogenesis [4].

The well studied organisms *Caenorhabditis elegans* and *Drosophila melanogaster* do not regenerate nervous system structures or display adult neurogenesis. However, some insects with longer lifespans, such as crickets, have centers of adult neurogenesis [6]. Planarians can regenerate almost complete nervous systems from multipotent neoblasts [7], [8]. Finally, it has been shown that some colonial tunicates can regenerate their whole body from multipotent cells [9], [10].

*Ciona intestinalis* (hereafter referred to as *Ciona*) is a tunicate deuterostome, and is thus a member of the clade corresponding to the closest extant relatives of vertebrates [11], [12]. Its simplicity and chordate characters have made *Ciona* one of the most widely studied invertebrate deuterostomes, particularly from a developmental point of view (http://genome.jgi-psf.org/Cioin2/Cioin2.home.html; http://hoya.zool.kyoto-u.ac.jp/cgi-bin/gbrowse/ci; http://crfb.univ-mrs.fr/aniseed/index.php).

Following the sequencing of two *Ciona* genomes, facilitating the characterization of regulatory elements, and improvements in methods for raising tunicates in the laboratory, many lines of transgenic animals expressing fluorescent markers in a wide array of patterns under the control of endogenous or exogenous promoters have been generated and are available [13], [14].

**Figure 1:**
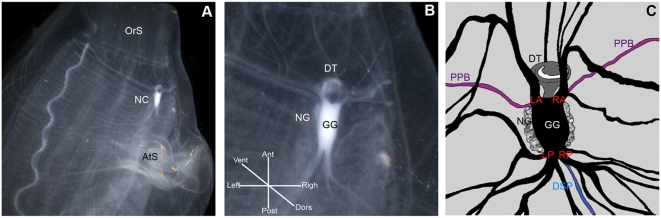
Ciona neural complex anatomy. A Neural complex (NC) of a wild-type animal, located between the oral siphon (OrS) and the atrial siphon (AtS). B Higher magnification of the neural complex (NC) composed of the neural ganglion (GG), the neural gland (NG) and the dorsal tubercle (DT). C Nomenclature used to name the nerves emerging from the ganglion. Peripharyngeal band (PPB), left anterior nerve (LA), right anterior nerve (RA), left posterior nerve (LP), right posterior nerve (RP), dorsal strand plexus (DSP).

The tunicate nervous system comprises the cerebral ganglion, the nerves of the body wall, the visceral nerve, the dorsal strand plexus and sensory structures connected to the siphons [15], [16]. The neural gland, which connects to the pharyngeal lumen via the ciliated duct and the ciliated funnel (partially filled by the dorsal tubercle) is located close to the ganglion. Together, these organs form the neural complex (Fig. 1). It has recently been suggested that the neural gland and ciliated duct may be homologous to the circumventricular organs (e.g. the choroid plexus) of vertebrates, with a putative role in controlling the homeostasis of the fluid surrounding the neural complex [17], [18] The cerebral ganglion includes most of the neural cell bodies of the central nervous system (CNS). Nerves leaving from the ganglion innervate the siphons, body wall and caudal viscera.

Following ablation of the entire neural complex, animals appear normal and continue to filter water and to feed. However, this ablation has some detectable effects, such as altered behavioral responses to stimulations. Squirting, consisting of retraction of the whole body when one of the siphon tips is touched or the crossed siphon reflexes when the tentacles are touched are among the responses that are impaired (reviewed in [19], [20], [21]).

CNS regeneration in *Ciona* has been studied in detail since its discovery by Schultze (1899). Following complete ablation of the neural complex, including part of the pharynx and the body wall, the neural complex regenerates completely within about a month [22]. Bollner *et al.* have shown that the expression patterns of certain transmitters (i.e. GNRH, 5-HT, SP) are recovered in the new cerebral ganglion, which, however, remains smaller than the original [23], [24].

The rate of regeneration in an animal depends on many factors, including temperature, food, stress, and the environment [25]–[27]. Some studies in echinoderms have indicated that the proportion of tissue lost is also an important regulator of the rate of regeneration [28]. It has been shown in vertebrates that young individuals have higher regenerative capacities [29], [30]. However, no systematic investigation has addressed the correlation between size and regeneration in ascidians.

We analyzed the regenerative process with a high level of temporal, morphological and functional resolution, by live imaging and functional analysis of the same animals over the course of one month of regeneration. We also compared the rates of regeneration in regenerating animals of different sizes. We used transgenic animals to achieve a higher cellular resolution of the regenerating nerves. This made it possible to show that proliferation occurred at the tip of the nerves during regeneration, consistent with each nerve having a regenerative blastema.

## Results

### Wildtype regenerative stages

We divided regeneration into stages, to obtain a clearer view of the complex temporal and spatial changes occurring during this process. Regeneration has previously been divided into stages on a purely temporal basis (i.e. first week, second week, etc. [22], [31]). However, regeneration speed varies considerably and is particularly sensitive to rearing conditions (e.g. temperature), resulting in imprecise staging. We identified and rigorously defined four different stages of morphological regeneration: *healing* (I), *nerve merging* (II), *structural regeneration* (III) and *functional regeneration* (IV).

**Figure 2:**
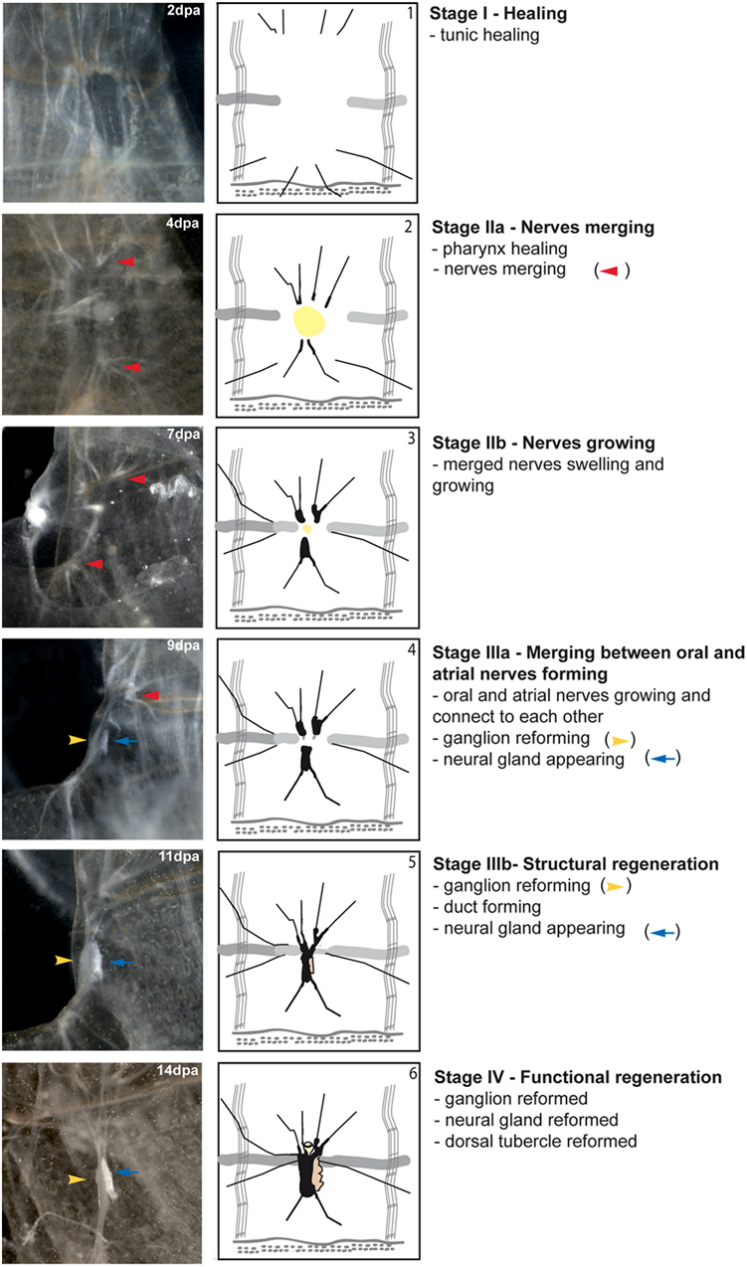
Visualizing the stages of regeneration. The first column shows the various stages of the regeneration process in a single middle-sized animal. The second column shows the regeneration process in a small animal. The third column provides a short description of the main features of the different stages of the process. Stereomicroscope images taken with the Leica MZFLIII (∼8×).

Figure 2 illustrates this generalized regenerative process, based on the analysis of more than 100 regenerating neural complexes. All images were obtained after carefully opening the tunic and photographing the regenerating neural complex from the outside through the body wall. A description of the events taking place during each stage is provided in Table 1 Characters and Stages.

**Table 1:** Stages of regeneration and characters.

Stages of regeneration and included characters	Description	Timing (dpa)
**Stage I**		**3.6±0.3**
Tunic healed	The hole in the tunic is closed	3.6±0.3
**Stage II**		**8.3±0.8**
Anterior nerves merging	The two anterior nerves merge	3.6±0.3
Posterior nerves merging	The two posterior nerves merge	3.8±0.3
Posterior thickening	There is a swelling of the posterior nerve endings	6.2±0.2
Anterior thickening	There is a swelling of the anterior nerve endings	6.6±0.2
Pharynx healed	The pharynx is closed	7.4±0.6
**Stage III**		**14.5±2.7**
Posterior structure	Merged nerve thicken and form a structure	8.8±0.2
Lateral net	Lateral connections between the anterior nerve endings	9. 6±0.2
Posterior horns	Nerves extending anteriorly from the posterior structure	11. 2±0.7
Anteroposterior connection	Visible neural connection between anterior and posterior, C→B transition	14. 0±0.8
**Stage IV**		**18.4±0.3**
Anteroposterior fusion	Connections between anterior and posterior are reinforced	16.2±0.2
Ganglion	A thin ganglion has formed	16.8±0.7
Dorsal tubercle	A dorsal tubercle is visible	17.8±0.3
Gland	A gland is visible	18.3±1.0

#### Stage I “*healing*”

This stage was characterized by the closing of the ablation wound by the tunic. A thicker transient tissue was initially formed. Complete tunic healing then occurred, with the new tunic becoming indistinguishable from the normal tunic (Fig. 2).

#### Stage II “*nerve merging*”

Soon after ablation, the sectioned nerves began to extend towards the wounded area. Adjacent nerve ends merged into networks with a higher density of regeneration foci in posterior than in anterior areas. The ends of the nerve fibers then thickened. We defined stage II “*nerve merging*” as the time at which the main branches of each of the four paired nerves could be seen to have merged in the regenerating area (Fig. 2). The healing of the pharynx took place during this stage and may be defined as a process in which the edges of the pharyngeal wall around the hole and epithelium resulting from the ablation procedure drawn together to close the wound. We defined “*pharynx healing*” as the stage at which the pharynx wound was closed, rather than the stage at which all muscles were repaired and in place. The timing of this process differed between individual animals.

#### Stage III “*structural regeneration*”

This stage was defined by the completion of four characters, all pertaining to neural structures:

1. The anterior nerves grew and sent out fine fibers in posterior and lateral directions. These fibers frequently formed a loose net-like structure of connecting lateral nerve endings (referred to here as the lateral net). More nerves extended in a posterior direction from the anterior network.
2. The two paired posterior nerve endings, lying next to each other, simultaneously thickened and formed a posterior structure.
3. This posterior structure then sent out nerves in an anterior direction.
4. The fine anterior and posterior nerve ends met and their connection defined the completion of stage III. It is this connection of anterior and posterior nerves that is responsible for the partial recovery of behavior as seen by the C→B transition described below.

#### Stage IV “*functional regeneration*”

This stage was defined as the thickening of existing anterior and posterior connections, followed by the recovery of all neural complex structures: a new small ganglion, a new gland and a new dorsal tubercle. It is during this stage that the B→A transition occurs reestablishing normal function (see next section: Functional recovery and physiological reflexes).

Observation under the dissecting microscope showed that all the neural structures were present at stage IV, although they were all slightly smaller than normal. We cannot exclude the possibility that changes to the morphology of the nervous system, such as the rewiring of interneuronal connections, took place. However, it was not possible to characterize such changes under the stereomicroscope.

### Functional regeneration/recovery and physiological reflexes

**Figure 3:**
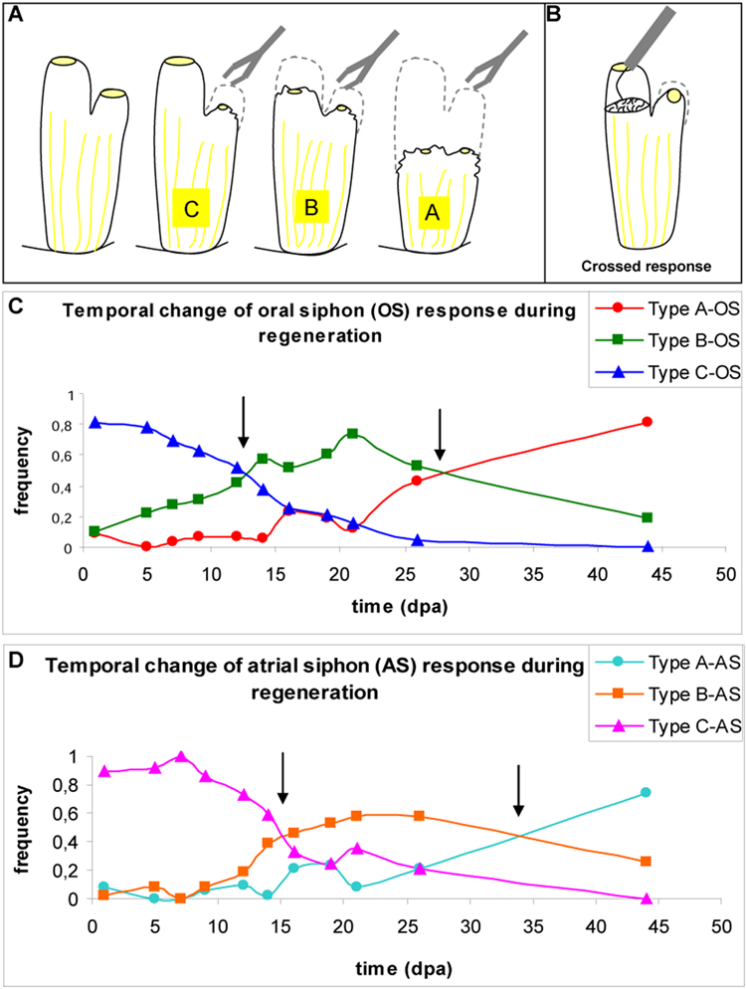
Functional regeneration assessed with the siphon stimulation test (SST). A Reflexes described in this paper in response to SST: type A, type B and type C. B Tentacle stimulation test (TST). Recovery of the reflex response of oral (C) and atrial (D) siphons. Frequency of each reflex response over time. The non ablated reflex response is characterized by ∼80% type A and 20% type B reflexes. Arrows indicate the transitions from type C–>B and type B–>A.

Based on the work of Mackie *et al.* (2006), we devised two behavioral tests. **The siphon stimulation test** (SST) involved gently touching the rim of either the oral or atrial siphon with large blunt forceps (Fig. 3a). Two types of response were observed in intact animals. Most animals retracted the whole body (type A response, also known as the squirt response), whereas some animals retracted both siphons synchronously without retracting the whole body (type B response) (Fig. 3a and Supp. Video S2). Intra-individual variation was observed, with a type B response sometimes observed in animals normally giving a type A response. Differences were also sometimes observed between the response to stimulation of the oral and atrial siphons.

Ablated animals were allowed to recover for one day and their reflexes were then tested. A third type of reflex response never seen in normal animals was then observed. This response consisted of the retraction of the stimulated siphon only (type C response) (Fig. 3a and Supp. Video S2). Animals in these conditions were much less reactive. During the regenerative process, type C responses gradually disappeared with time and the type A response was re-established. Type B responses seemed to be an intermediate, observed transiently in regenerating animals. Another transient state was also observed: a type C response followed by the slow, delayed closure of the non stimulated siphon. These observations allowed us to establish a framework for monitoring functional regeneration in *Ciona*.

We carried out the SST on the regenerating animals used in the morphological study described above, and scored changes in the frequencies of the different types of response as regeneration proceeded. The frequency of each response was calculated following the stimulation of both siphons and was plotted against time (Figure 3).

The transition from one response to another (C→B and B→A) was identified as the intersection point of the two response curves. For example, the C→B atrial transition occurred at 13 days post ablation (dpa) for medium-sized animals and 19 dpa for large animals, indicating that size played an important role in determining the rate of reflex recovery. The C→B transition required the completion of stage III. This morphological stage includes the first rejoining of nerve endings, supporting the intuitive conclusion that this connection is required to link the response of the two siphons. The B→A transition occurred later, when the morphological connection was strengthened and integrated during stage IV. Once again, the formation of a ganglion can be linked with the recovery of a pattern of behavior that had been lost.

The **tentacle stimulation test** (TST) is a variant of another test known as crossed response stimulation. Mackie *et al.* 2006 used this method to provoke the partial closure of the atrial siphon following stimulation of the tentacles in the oral siphon (Fig. 3b).

The tentacle stimulation test was more difficult to perform than the SST and was used principally to illustrate another type of functional regeneration/recovery. The TST involves placing an ablated animal next to a non ablated animal and comparing the responses of the two animals to similar stimulations. If the tentacles in the oral siphon were touched without touching the siphon wall, the atrial siphon closed halfway and then opened again, as described in detail by Mackie *et al.* (2006) (Supp. Video S3). In our test, we allowed the animals to lie on their sides and inserted a very fine needle (Wolfram 0.1 mm diameter) into the oral siphon to stimulate the tentacles. In normal animals, the crossed reflex was systematically observed, but only after an average of two attempts. Touching the wall or other parts of the siphon led to a type B response, as described above. Some of the regenerating animals displayed a crossed response after regeneration, but we were unable to determine an average time point for this test, as it took place later than stage IV. We conclude that the TST can be used to estimate the functional endpoint of regeneration.

### Rate of Regeneration as a function of morphology and size

**Figure 4:**
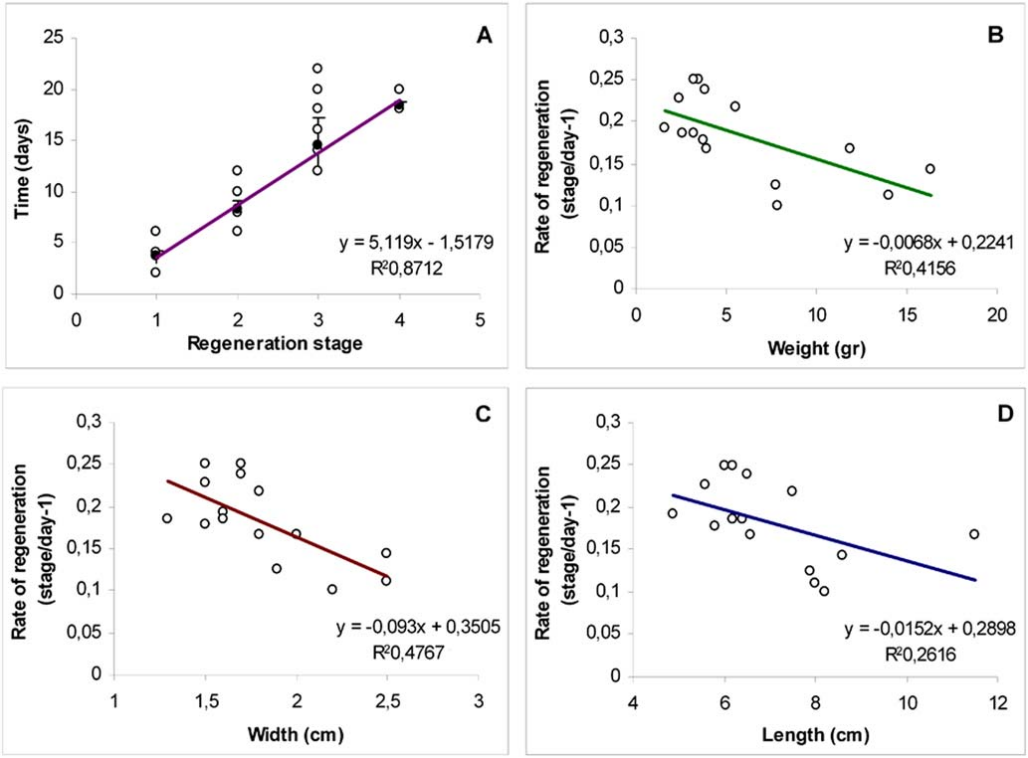
Regeneration is inversely proportional to size. A Relationship between time (in days) and regeneration stage. Stages are defined such that they have a similar duration. Mean stage duration (or regeneration rate) can then be calculated as the correlation coefficient for the linear regression. B–D Significant (p<0.01) linear relationship between different morphometric parameters and rate of regeneration (stages/day). B, Length (in cm); C, width (in cm); D, weight (g).

The four stages of regeneration had similar time courses (Table 1). The regeneration rate was estimated for each individual as the correlation coefficient for the linear relationship between stage and time (Fig. 4a). Large differences were observed between individuals. These differences may be partly accounted for by differences in the size of the animals. A negative correlation was observed between three morphometric parameters (length, width and weight) and regeneration rate (Fig. 4b–d), with larger animals regenerating more slowly (i.e. each stage lasted longer) than smaller animals.

### GFP gene expression in neural structures of the E[MiTSAdTPOG]15 (E15) line

**Figure 5:**
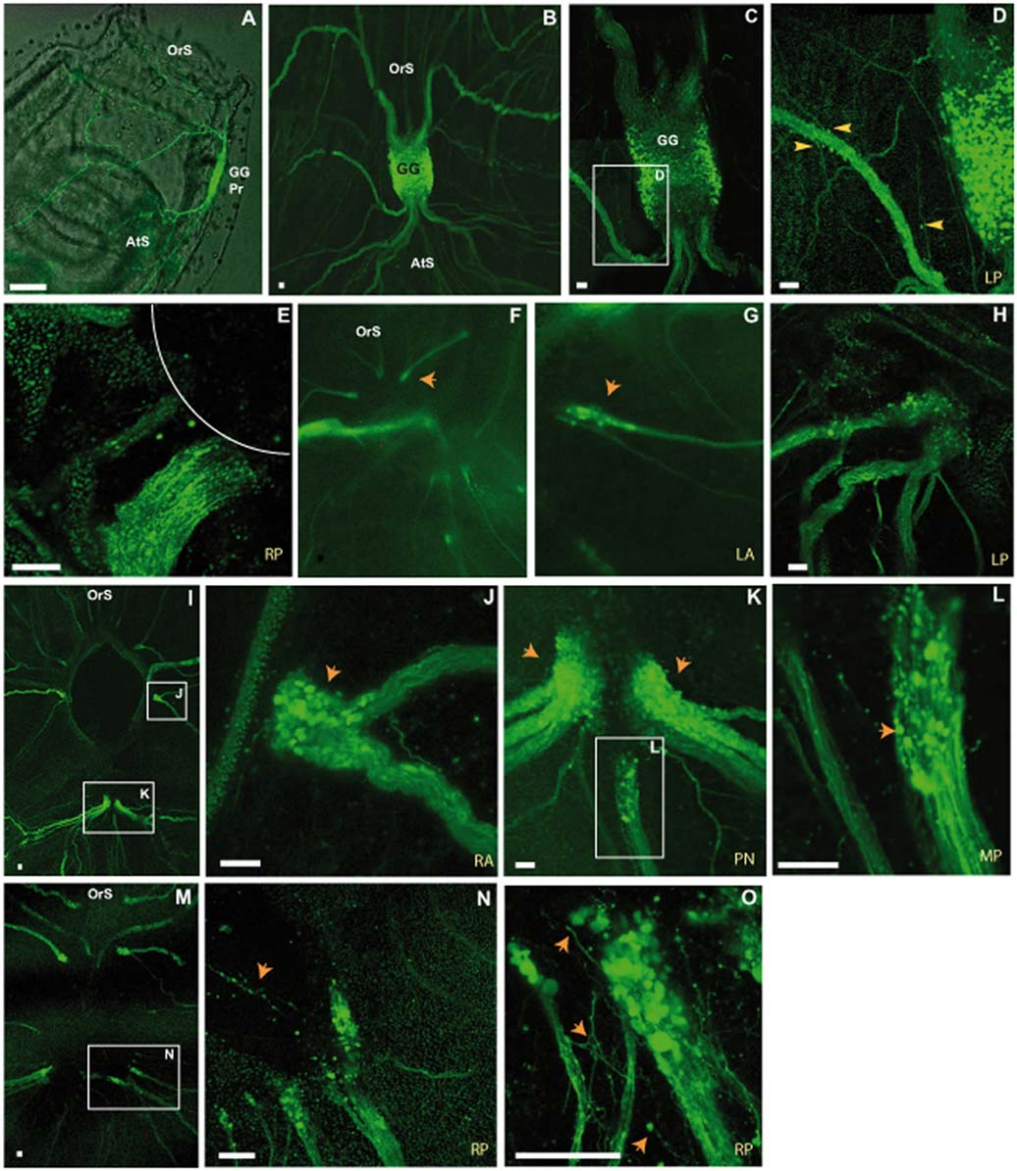
Cell masses arranged around nerve ends. Nerves visualized in E15 transgenic animals. GFP is produced only after the metamorphosis of juveniles (A). Ganglion and emerging nerves in B. Nerves in close proximity to the ganglion before ablation. C and D show peripheral cell bodies (yellow arrowheads). Nerve ending just after ablation in E, with line indicating hole. At 2 dpa (stage I) no clear pattern of nerve cell bodies is observed at the nerve endings (F, G and H). Nerve ending with cell bodies forming a swelling at 4 dpa (I, arrowheads in J and arrowheads in K). Some thickened nerve cell masses start to extend their axons toward the anterior at 4 dpa (L). At 9 dpa (early stage III) the nerve end cell masses decrease in size and send lots of extensions in the anterior and posterior directions, sometimes making connections (M, arrowheads N, and O). All scale bars, 50 µm. Ganglion primordium (GG Pr), atrial siphon (AtS), oral siphon (OtS), left posterior nerve (LP), right posterior nerve (RP), mid-posterior nerve (MP), posterior nerves (PN), right anterior nerve (RA) and left anterior nerve (LA). Fluorescent confocal images obtained with the Leica SP5 system (5×, 20× and 40×).

We used the transgenic lines to investigate in more detail the cellular aspects of regeneration. We used the E15 line, which expresses a GFP transgene in nerve cells (Awazu *et al.*, 2007). GFP was detected in known neural structures, such as the cerebral ganglion, the exiting nerves and the peripharyngeal band. We analyzed GFP gene expression in the E15 line during development and found that this gene was expressed from metamorphosis onwards. The developing ganglion, the neurites around the primordial siphons and the branchial basket displayed strong GFP gene expression (Fig. 5a).

**Figure 6:**
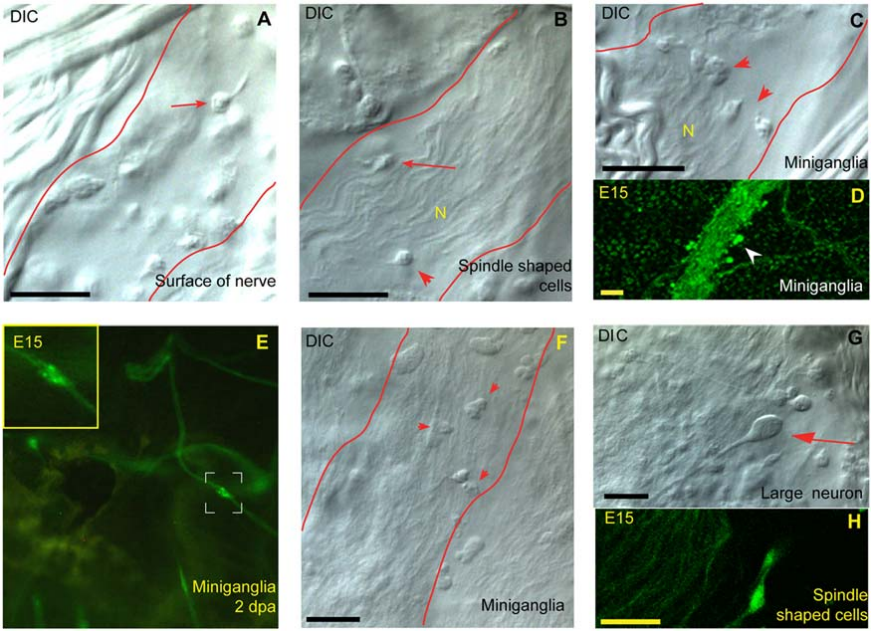
Nerve cell types found peripherally and “miniganglia”. Neural cells located peripherally to the ganglion along the major nerves (red line indicating nerve tract). These are either small round cells with a clear neuritic extension (arrow in A) or spindle-shaped cells (arrowhead in B) integrated into the nerve fibre (N). The round cells are sometimes organized into “miniganglia” (arrowheads in C, D and E). E shows a “miniganglion” soon after ablation (2 dpa, inset showing close-up). Other cells along the peripheral nerve (arrowheads in F). G shows the different morphology of the larger motor neurons seen only in the ganglion (arrow). H shows some of the spindle-shaped cells in the area just adjacent to the ganglion. Scale bars, 20 µm. Fluorescent confocal images obtained with the Leica SP5 system and DIC images obtained with the Leica DMRBE compound microscope (40×).

At least four cell types in the adult ganglion of the E15 transgenic line expressed the transgene. One was characterized by cell bodies up to 20 µm in diameter, mostly corresponding to the previously described large motor neurons (Fig. 6g) [32]. There was also a population of medium-sized (∼13–14 µm) neurons, with a similar morphology to the larger ones. The medium-sized neurons were located in the cortex of the ganglion, but also in the posterior roots. Those located in the posterior roots extended towards the ganglion (See Supp. Videos S5 and S6). Smaller (∼7 µm) spindle-shaped bipolar neurons and “rectangular” 10 µm cells were also observed (Fig. 6h). Multipolar neuronal cells were found but were often difficult to distinguish from the “rectangular” cells.

The transgene was expressed in all nerves in the E15 line. The dorsal strand plexus, extending in the dorsal and posterior directions from the right medial nerve root, was also GFP-positive (Supp. Videos S6). The visceral nerve, exiting as a medial branch from the left posterior nerve, was also labeled (Supp. Video S6). As in the juvenile, we were able to follow neurites some distance from the ganglion, suggesting that GFP was present in axons. Furthermore, we were able to follow closely the single axons of the large motor neurons. Thus, it seems that all the neural cells investigated in the E15 line expressed the GFP transgene.

Unexpectedly, we found that the cerebral ganglion was surrounded by a very diffuse network of neurites (Fig. 5d). The cell bodies of this nerve network were located outside the ganglion and the network was connected to the main exiting nerves (Fig. 5d, arrowheads). Further away from the ganglion, there were nerves that had split into several branches, and most, if not all of the major nerves were also associated with several cell bodies (Fig. 6). The exact nature of these cells remains unknown, but there appeared to be at least two different populations. The first population was not integrated into the nerve, but was located on the border of the main nerve fibers, with neurites extending into the nerve (Fig. 6a arrow). These nerve cells were sometimes organized into mini-ganglia of a few cells (Fig. 6c–e). The second population comprised cells integrated into the nerves and had a bipolar spindle-shaped form (Fig. 6b arrows). These cells were small and we observed no large neuronal cell bodies outside the ganglion in the non ablated animals (See figure 6).

### Early regenerative events visualized in transgenic lines (Fig. 5)

We visualized early regenerative events, using transgenic animals and confocal microscopy. Examination under the dissecting microscope showed that the general course of regeneration in transgenic animals was similar to that observed in wild-type animals.

Using the E15 line, we were able to describe the initial steps of regeneration by visualizing, soon after ablation, single GFP-positive cells. We concluded from stereomicroscopy studies that new structures originated from the residual neural structures on all sides of the ablation area, not only from the posterior regions containing the dorsal strand. We decided to focus our attention on individual regenerating nerve endings, following their regeneration by confocal microscopy in transgenic animals. Before ablation, the nerves were not thickened, and only some of the nerves had GFP-positive cell bodies close to the area of the intended lesion. Immediately after ablation, we identified axonal material, defined by the accumulation of GFP released from the nerve endings. At stage I (2 dpa, 14°C), the nerve endings were slightly thickened and a few cell bodies positive for the transgene were observed among the other negative cells (Fig. 5f–h). At the start of stage II (4 dpa, 14°C), the nerve ending was thickened and a mass of cells formed, many of which were GFP-positive. At this stage, we first observed larger (10 µm) cell bodies, but no neurons could be clearly identified (Fig. 5i–l). All the nerves, including the lateral and anterior ones that had been cut showed this pattern of regeneration. At the start of stage III (9 dpa, 14°C), the cell mass began to change, with some cell bodies being left behind and axons and cells being sent out centrally towards the other nerves, even extending into newly formed tissues (Fig. 5m–o).

Time-lapse analysis of the regenerating nerve endings showed extensive path-finding activity, with neurites and growth cones forming and collapsing (Supp. Video S4). Neural cells also joined from lateral sides of the regenerating nerves.

### Blastema formation

**Figure 7:**
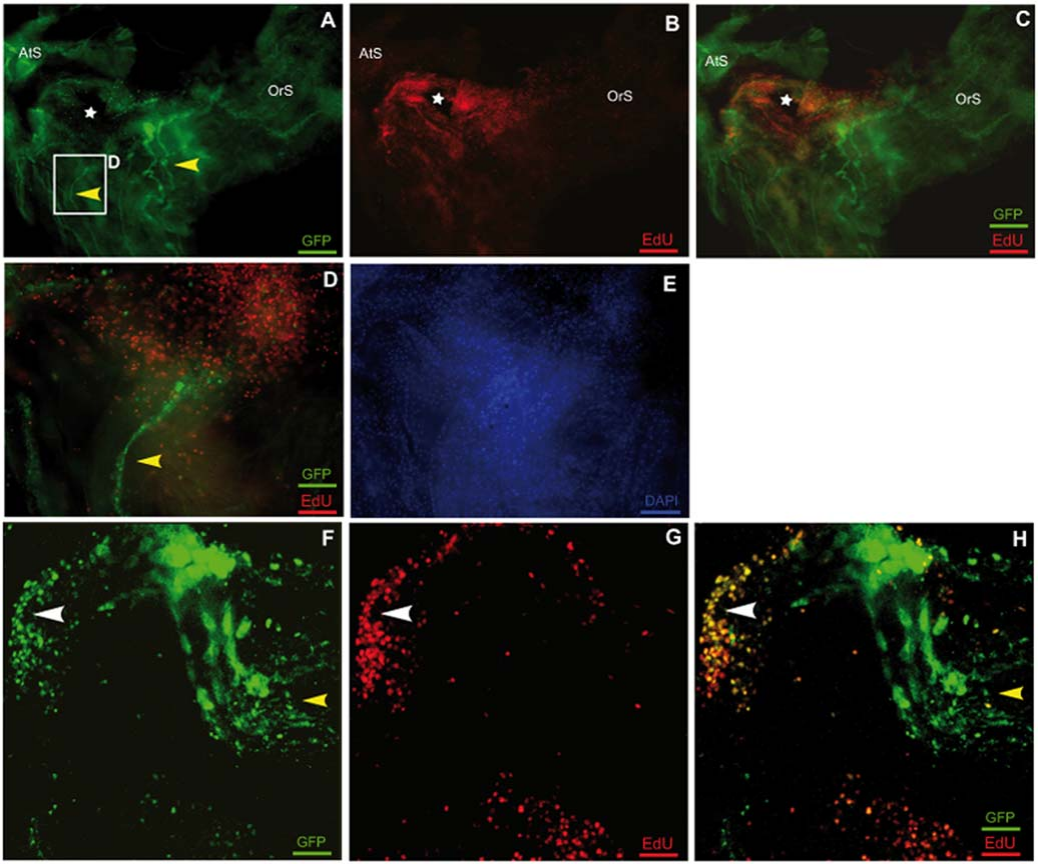
A blastema formation is indicated by the zones of proliferation bordering the regenerating nerves. A–C Whole-mount preparation of an E15 transgenic animal (5×, apotome) at 4 dpa. Staining for GFP staining is shown in green and corresponds to the nerves (yellow arrowheads) with EdU staining visualized in red. A and B, merged in C. The ablation area is indicated with a white star. D, E High magnification of a nerve ending (20×, apotome). The merged image panel in D shows a zone of dense proliferation in the continuation of the nerve. DAPI staining in E, showing that the proliferative and the non proliferative zones have similar cell densities. F–H High magnification of a nerve ending (63×, projection of a stack of confocal images). A proliferation zone can be seen around the nerve ending and in its continuation. In the nerve ending, a double-stained area (GFP and EdU) was observed, demonstrating the presence of a neurogenic blastema (white arrowhead).

We investigated the role of proliferation in the early stages of regeneration, by labeling the regenerating transgenic animals with the modified nucleotide EdU. Like BrdU, EdU is incorporated into the nuclei of mitotic cells during S-phase. Several prominent zones of proliferation were observed after incubation in 200 µM EdU for two days, two days after ablation (Fig. 7). As expected, the area immediately around the hole displayed high levels of proliferation. Adjacent to the nerve endings there was a small zone of unlabeled cells. However, at the periphery of these negative cells, there was a group of double-labeled cells, which both expressed the transgene and incorporated EdU, indicating recent cell division (Fig. 7j). The presence of these cells indicates that a blastema was forming at the tip of each nerve, supplying nerve cells for reformation of the nerve network.

## Discussion

We provide here a biotechnological framework for studies of central nervous system regeneration in the tunicate *Ciona*. We subdivide the process into four different stages and provide a comprehensive spatial and temporal description of neural complex regeneration. We also describe the development of techniques for studying events in live cells in tissue explants, the study of functional regeneration and real-time imaging of the process using transgenic animal lines. Finally, we provide evidence that regeneration occurs around each regenerating nerve ending.

*Ciona* CNS regeneration studies have been largely based on the work of Bollner and coworkers, who showed that immunoreactivity for GNRH, 5-HT, and SP recovered a pattern similar to that seen in normal adult, which they considered to be an indicator of full functional recovery. However, there are no physiological studies validating this inference [23], [31]. We therefore decided to investigate physiological reflexes in adult and regenerating *Ciona*.

The reflexes and responses we identified in *Ciona* are essentially the same as those elicited in *Corella sp.* and *Chelyosoma productum* in the experiments performed by Mackie and co-workers [20], [21]. All physiological experiments were performed on whole animals, with simple procedures, and enabled us to follow most of the responses reported in previous studies, except for velar minimal contraction and ciliary arrest, which were not studied ([21]). However, *Corella* and *Chelyosoma* do not regenerate a complete neural complex. We therefore decided to study regeneration in *Ciona* regeneration, as we did not wish to extrapolate between species.

The simple siphon stimulation test (SST) generated up to three sequentially separable reflexes during CNS regeneration. Investigation of the transitions between these reflexes over time, at a high temporal resolution, provides a precise tool for helping stage identification. Our studies of the temporal recovery of physiological reflexes also open up new possibilities for more detailed studies of the effects of temperature, food, stress and genetic pathways on regeneration. The reflexes tested are easy to study and change considerably in response to ablation. The sequential nature of the recovery makes it possible to combine morphological and/or ecological tests with behavioral tests thereby linking parameters of qualitatively different characters and making the *Ciona* model very tractable.

We provide evidence that regeneration rate is dependent on size. Smaller animals regenerate faster than larger animals, as also shown by the functional study. Bollner defined adults as animals more than 50 mm long, all of which had mature gametes [31]. Lender and Bouchard-Madrelle (1964) provided no information about the size or age of the animals they used, but we can assume that they were adults. The ablation procedure is easier to perform on larger animals, so large adults were probably used in these studies. It is difficult to estimate the age of wild-type *Ciona*. As stated above, size differs both within and between age groups. *Ciona* settle throughout the summer season (May to September, Swedish west coast), with fecundity peaking in June. They live for 12 to 18 months and retain their ability to regenerate throughout the summer season. In warmer waters, they are reported to spawn throughout the year. In our conditions, regenerative capability decreased in late fall, probably because of lower water temperatures, lower algae concentrations and the aging of the individuals.

Few studies have taken size into account as a factor affecting regeneration or growth. In most studies, this issue has been avoided by comparing animals of similar size. In a study of regeneration in brittlestars, size did not affect the rate of regeneration [28]. Given the extensive variation in regeneration rate observed in *Ciona*, particularly as a function of temperature, we investigated the effects of size on regeneration rate in more detail. The reason for the inverse correlation between size and regeneration rate is unknown, but may have more to do with age than size. However, it was not possible to test this hypothesis, because we could not determine the age of animals collected in the wild. It should be possible to overcome this limitation by using laboratory-reared animals of the same age, but reared under different conditions (e.g. rich or poor food regimen) and therefore differing in size. By comparing such animals, and animals of the same size but different ages, it may be possible to determine which of these two factors is the most important.

Our detailed stereomicroscopy analysis has provided insight into the kinetics of neural complex regeneration in *Ciona* [22], [31]. Using transgenic animals and confocal microscopy, we were also able to study in detail various cellular aspects of regeneration. Nerve regeneration starts soon after ablation, and thickened nerve endings soon extend and join adjacent neural structures. We have shown that cells accumulate around the severed nerve endings. Remarkably, this occurs not only from the posterior side, but from all around the ablation area. Thus, each severed nerve ending has the potential to organize new structures, which later establish connections with other adjacent nerve endings. Using time-lapse imaging and explants, we showed that these cells extended axons and growth cones toward other nerve endings. We also confirmed observations of the sprouting of fibers from nerve cell masses and observed cell bodies leaving the mass of cells and following the fibers at a later stage of regeneration (Fig. 5).

Our findings revise the former view that no neuronal cell bodies are associated with the nerves outside the ganglion. Indeed, we observed neural cell bodies along the nerve in areas organized into mini-ganglia, together with a loose network adjacent to the ganglion. Some of these cells are probably similar to those described by Koyama in electron microscopy studies of *Polyandrocarpa* [33]. However, no large neural cell bodies were visible anywhere other than in the ganglion in this previous study. Difficulties visualizing the small neurites extending from these neural cells probably account for these cells being overlooked in previous studies. This problem was resolved by our use of the GFP transgenic line.

It remains unclear where the new cells accumulating at nerve endings come from. Consistent with the observations of Bollner and coworkers (1997), we observed extensive proliferation in the dorsal strand (data not shown). This structure is clearly a possible source for the cells contributing to regeneration. However, we also found that nerve cells formed at the edge of the regenerating nerve ending and identified a potential “blastema”. The blastema is a regenerative unit (analogous to the developmental bud) composed of a mass of undifferentiated cells capable of forming regenerative tissue. This structure has been found in most cases of regeneration, although its form is highly variable [7], [34]–[36].

The presence of a blastema at anterior nerve endings suggests that the new nerve cells of the regenerating ganglion are unlikely to be derived from the cells of the dorsal strand or the associated plexus. Indeed, these structures are much further away. We cannot rule out the possibility that undifferentiated cells, such as the pluripotent cells in planarians or colonial tunicates, migrate and turn into neurons. However, morphologically, nerves seem to reform or at least to steer the reformation of their own network [8], [10].

Our confocal time-lapse microscopy studies detected no movement of GFP-positive cell bodies along the axons toward the cut nerve during the first 36 h. Thus, although further studies are required to determine more definitively the source of the cells present in the nerve cell mass, we believe that the blastemal formation we describe here is the most likely source of the cells involved in regeneration of the neural ganglion. However, the cellular composition of the blastema remains unknown.

We present here a model suitable for use in further studies of *Ciona* CNS regeneration. We hope to have provided a strong basis, extending from description of the physiological response to cellular events and the associated key morphological stages.

In vertebrates, only neural structures displaying continuous adult neurogenesis seem to be capable of regeneration (the visual system of teleostean fishes is a case in point). However, it has recently been shown that zebrafish spinal cord can not only regenerate motor neurons but also produce new ones in response to lesions in areas that are not normally neurogenic [3]. *Ciona* is a chordate, like other efficient regeneration models such as salamanders and fish, and is more distantly related to other models, such as planarians. However, *Ciona* regeneration differs from vertebrate regeneration in several ways. In particular, the machinery for myelin formation is entirely absent, so the nerves of ascidians, like those of agnathans, are unmyelinated [37]. By contrast, tunicates have three layers of nerve encapsulation, as described in detail for *Polyandrocarpa misakiensis* [33]. These differences in nerve environment clearly lead to different regenerative strategies in these species.

The *Ciona* model is a promising tool for addressing questions concerning regeneration in general or specific genetic questions in a functional context. Tools provided by the two *Ciona* genome projects, which, together with genomic condensation, make promoter analysis easier and should also make it easier to address functional genetic questions. *Ciona* is already a major model organism for developmental biologists. These aspects, together with the high degree of transparency (particularly for laboratory-reared animals), short generation time (3 months) and ease of culture and ablation, also make *Ciona* an ideal candidate model of regeneration. However, one potential problem with the use of this model is the apparently polymorphic background in *Ciona*, making genetic investigations more complex than those in models with constant genetic backgrounds, such as *C. elegans*. Further studies using the methods described here should document regeneration in the diverse *Ciona* populations found worldwide.

With the advent of UV-convertible reporter genes, such as KAEDE [38] and the probable construction of inducible genetic lines in *Ciona*, we will have tools that, together with these findings, will make it possible to address questions relating to cell fate and lineage and the function of unknown genes. This work helps to establish *Ciona* as a neural regeneration model complementary to limb and tail regeneration in salamander and zebrafish brain ablation [3], [39].

## Materials and Methods

### Culture of adult *Ciona* during regeneration

Wild-type *Ciona* were collected at various locations in the vicinity of Kristineberg Marine Research Station, Fiskebäckskil, Sweden. All animals were kept in running seawater from the Gullmarsfjord taken from 5 meters below the surface (July–August 2007). Salinity varied over the study period, from 21 to 29 PSU, with a mean value of 24. The temperature of the water varied over the period, from 16 to 20.5° C, with a mean value of 17.5° C. Approximately 20 animals were kept in an 18 liter aquarium, with a water flow rate of ∼2 l/ min. No additional food was supplied. The animals were retained in suspended hard net baskets within the aquarium, to separate them from fecal material. Tanks were cleaned by siphoning every two days.

Adult transgenic animals were kept in the same way as wild-type animals, but with a lower water flow rate, necessitating daily feeding with *Rhodomonas spp.*. Experiments were also performed later in the season (September–October), when salinity varied from 22 to 29 PSU, with a mean value of 25. Water temperature over this period varied from 12.5 to 15.5° C, with a mean value of 14° C. All water in contact with transgenic animals was sterilized by UV irradiation and handled according to local regulations.

Individual animals were labeled by injecting a combination of colored drops of an elastomer paint (VIE, Northwest Marine Technology) under the tunic, close to the site of attachment.

### E[MiTSAdTPOG]15 promoter transgenic animals

Adult *Ciona* of the E[MiTSAdTPOG]15 transgenic line were obtained at the Shimoda Marine Research Center (Awazu et al., 2007). E15 expressed the GFP gene in most neurons.

### Ablation technique and anesthetic

Animals were anesthetized with MS222 (0.4 g/l) or propylene phenoxetol (0.06%) in seawater for 15 to 30 minutes before ablation, dissection or live imaging. Ablations were carried out with fine forceps and biopsy punch tools (2 and 3 mm diameter; Stiefel® biopsy punch; ablation technique described in Table S1 and visible in Supplemental. Video S1). By stabilizing the tissues with the forceps, we were able to remove the entire ganglion and the gland including the ciliated funnel and dorsal tubercle in a single action, minimizing trauma. The use of Sylgard®-coated Petri dishes (Sylgard® 184 Silicone Elastomer) was found to be essential. In our hands, this technique gave consistently better results than traditional microdissection. The complete sedation and opacity of animals also affected the likelihood of success. In the smallest individuals used, mortality levels were significantly higher than in controls that did not undergo ablation (<5%). This probably due to poor ablation and to smaller animals being unable to cope with repeated openings of the tunic. Mortality rates were so high in the smallest group that this group was excluded from the physiological reflex experiment. Previous experiments with other transgenic lines indicated that animals reared in Gif-sur-Yvette had more transparent tunics and mantles and were amenable to imaging without iterative tunic opening.

### Live imaging

For visualization of regeneration of the neural complex, we anesthetized individuals every other day, and analyzed and photographed the area of regeneration for each of the 30 animals of different sizes. The tunic was opened or cleaned when necessary, to facilitate these analyses, but we paid particular attention not to disturb tissues early in the healing process. The ability of *Ciona* to survive short periods without water and the possibility of repeated sedation made it possible to use live imaging with a dissecting microscope. This was important as it allowed us to follow regeneration in the same individual, at frequent intervals. Images were acquired with a Leica MZ 16 A stereomicroscope and LAS software.

### Nomarski images

Nomarski images were taken of small specimens of *Ciona*, which were sedated and then microdissected and mounted under a coverslip, fixed *in situ* with 4% PFA and then transferred through a series of glycerol solutions of increasing concentration, up to 100% glycerol.

### Confocal imaging of whole animals

Confocal imaging of regenerating whole *Ciona* was carried out principally with small individuals, with part of the semi-opaque tunic removed. The animals were placed under light pressure imposed by a small piece of glass, in a chamber with a coverslip at the bottom (Lab-Tek, NUNC). Images of fixed animals were collected with a Leica SP5 Laser Scanning Confocal Microscope and images of living animals were collected with multiphoton laser scanning microscope (MLSM). Animals were not harmed by the procedure.

### Explant preparation

Animals were sedated as described above, and prepared as described in supplementary material Table S1. The resulting glass-bottomed chambers (Lab-Tek, NUNC) with dorsal preparations were used for the imaging of live cells and for following cells by time-lapse confocal microscopy.

This procedure was used for all close visualization of nerve endings. The medium in the chambers was either filtered seawater (FSW) supplemented with 10 µg/ml kanamycin and 50 µg/ml gentamycin, or FSW supplemented with cell culture medium (L-15 Leibowitz, Sigma-Aldrich) and 50 µg/ml gentamycin. Tissue integrity, lack of symbionts, and closure of the ablation hole were used as indicators of a viable preparation.

### EdU incubations and staining for GFP

Newly proliferating cells in the early stages of regeneration were labeled by incubation in 200 µM EdU (Invitrogen) in 100 ml of seawater for 16 h. Incubations were performed two days after ablation. Whole-mount preparations were fixed by incubation with 0.4% PFA and washed in 0.1% Tween 20 in 1× PBS. They were stained with antibodies against GFP (1∶500 dilution of rabbit primary GFP antibody (A11122) and a 1∶200 dilution of mouse anti-rabbit secondary antibody coupled to Alexa 594 (A11037) from Molecular Probes). EdU was then detected with the procedure recommended by Invitrogen. We used Alexa Fluor azide 647 to detect the EdU-positive cells. Preparations were incubated for 15 minutes with a 1∶100,000 dilution of DAPI (Sigma, D9542) and rinsed in PBS.

### Behavioral responses

Wildtype *Ciona* were stimulated in their aquaria in a random order; first orally and, about 5 min later, atrially; without prior disturbance. The types of response were scored following two stimulations. To score a crossed reflex positive, the response had to be observed twice, in connection with a visual touch of the tentacle (see Supplemental Video S2). The experiments were always performed by one person stimulating the tentacle and another one scoring responses. In this way the crossed reflex is easily identified even though it is often followed by a type B response (see below).

### Rate of regeneration & Statistics

Regeneration rates were compared across individual sizes, by scoring the attainment of various structural characters at a given time point after ablation. Individuals were classified according to size. The “medium” group corresponded to animals with a mean length of 6 cm (max 7.5, min 4.5), a mean drip-dry weight of 3 g (max 5.5, min 1.5) and a mean width of 1.5 cm (max 1.8, min 1.1). The “large” group corresponded to animals with a mean length of 11 cm (max 15, min 7.9), a mean drip-dry weight of 16.7 g (max 25.9, min 7.7) and a mean width of 2.4 cm (max 3.3, min 1.8). The “small” corresponded to animals smaller than those of the preceding two groups and was omitted due to high mortality rates.

Each mean value is expressed with its standard error (mean±SEM). Analysis of variance (ANOVA) and t-tests were used to determine the significance of the observed differences between groups. A simple linear regression model was used to assess the type of relationship between variables. All statistical methods used (ANOVA, linear regression) assume that the data are normally distributed. Shapiro–Wilk's (1965) statistic (W) was used to check that the data were a random sample from a normal distribution. Analyses were performed with SAS/STAT® software (SAS Institute Inc., 1990)

## Supporting Information

Table S1 Preparation instructions
(0.03 MB DOC)

Video S1 Ablation procedure using a biopunch
(4.97 MB MOV)

Video S2 Siphon stimulation test: responses of type A, B and C following the stimulation of a siphon tip
(2.35 MB MOV)

Video S3 Crossed reflex: retractation of the atrial siphon following stimulation of the oral tentacles
(3.28 MB MOV)

Video S4 Migration of cell bodies and axons around a regenerating nerve at 4dpa
(24.54 MB AVI)

Video S5 Rotational view of the anterior part of the ganglion
(23.96 MB AVI)

Video S6 Rotational view of the posterior part of the ganglion
(24.48 MB AVI)

## References

[1] Moshiri A, Close J, Reh TA (2004). Retinal stem cells and regeneration.. Int J Dev Biol.

[2] Koster RW, Fraser SE (2006). FGF signaling mediates regeneration of the differentiating cerebellum through repatterning of the anterior hindbrain and reinitiation of neuronal migration.. J Neurosci.

[3] Reimer MM, Sorensen I, Kuscha V, Frank RE, Liu C (2008). Motor neuron regeneration in adult zebrafish.. J Neurosci.

[4] Kaslin J, Ganz J, Brand M (2008). Proliferation, neurogenesis and regeneration in the non-mammalian vertebrate brain.. Philos Trans R Soc Lond B Biol Sci.

[5] Zupanc GK (2006). Neurogenesis and neuronal regeneration in the adult fish brain.. J Comp Physiol A Neuroethol Sens Neural Behav Physiol.

[6] Lindsey BW, Tropepe V (2006). A comparative framework for understanding the biological principles of adult neurogenesis.. Prog Neurobiol.

[7] Cebria F (2007). Regenerating the central nervous system: how easy for planarians!. Dev Genes Evol.

[8] Agata K, Umesono Y (2008). Brain regeneration from pluripotent stem cells in planarian.. Philos Trans R Soc Lond B Biol Sci.

[9] Kawamura K, Tachibana M, Sunanaga T (2008). Cell proliferation dynamics of somatic and germline tissues during zooidal life span in the colonial tunicate *Botryllus primigenus*.. Dev Dyn.

[10] Rinkevich Y, Douek J, Haber O, Rinkevich B, Reshef R (2007). Urochordate whole body regeneration inaugurates a diverse innate immune signaling profile.. Dev Biol.

[11] Bourlat SJ, Juliusdottir T, Lowe CJ, Freeman R, Aronowicz J (2006). Deuterostome phylogeny reveals monophyletic chordates and the new phylum *Xenoturbellida*.. Nature.

[12] Delsuc F, Brinkmann H, Chourrout D, Philippe H (2006). Tunicates and not cephalochordates are the closest living relatives of vertebrates.. Nature.

[13] Awazu S, Matsuoka T, Inaba K, Satoh N, Sasakura Y (2007). High-throughput enhancer trap by remobilization of transposon Minos in *Ciona intestinalis*.. Genesis.

[14] Joly JS, Kano S, Matsuoka T, Auger H, Hirayama K (2007). Culture of *Ciona intestinalis* in closed systems.. Developmental Dynamics.

[15] Mackie GO (1995). On the ‘visceral nervous system’ of *Ciona*.. Journal of the Marine Biological Association of the United Kingdom.

[16] Millar R (1953). Ciona Liverpool.

[17] Deyts C, Casane D, Vernier P, Bourrat F, Joly JS (2006). Morphological and gene expression similarities suggest that the ascidian neural gland may be osmoregulatory and homologous to vertebrate peri-ventricular organs.. European Journal of Neuroscience.

[18] Joly JS, Osorio J, Aluni A, Auger H, Kano S (2007). Windows of the brain: Towards a developmental biology of circumventricular organs and other neurohemal organs.. Seminars in Cell & Developmental Biology.

[19] Bullock TH, Horridge GA (1965). Structure and Function in the Nervous System of Vertebrates.

[20] Mackie GO, Burighel P, Caicci F, Manni L (2006). Innervation of ascidian siphons and their responses to stimulation.. Canadian Journal of Zoology.

[21] Mackie GO, Wyeth RC (2000). Conduction and coordination in deganglionated ascidians.. Can J Zool.

[22] Lender T, Bouchard-Madrelle C (1964). Études expérimental de la régéneration du complex neural de *Ciona Intestinalis* (Prochodé).. Bull Soc Zool.

[23] Bollner T, Beesley PW, Thorndyke MC (1992). Pattern of substance P- and cholecystokinin-like immunoreactivity during regeneration of the neural complex in the ascidian *Ciona intestinalis*.. Journal of Comparative Neurology.

[24] Bollner T, Beesley PW, Thorndyke MC (1997). Investigation of the contribution from peripheral GnRH-like immunoreactive ‘neuroblasts’ to the regenerating central nervous system in the protochordate *Ciona intestinalis*.. Proceedings of the Royal Society of London Series B Biological Sciences.

[25] Gunnarsson JS, Granberg ME, Nilsson HC, Rosenberg R, Hellman B (1999). Influence of sediment-organic matter quality on growth and polychlorobiphenyl bioavailability in Echinodermata (*Amphiura filiformis*).. Environmental Toxicology and Chemistry.

[26] Candia Carnevali MD, Bonasoro F, Patruno M, Thorndyke MC, Galassi S (2001). PCB exposure and regeneration in crinoids (Echinodermata).. Marine Ecology Progress Series.

[27] Petersen JK, Schou O, Thor P (1997). *In situ* growth of the ascidian *Ciona intestinalis* (L.) and the blue mussel *Mytilus edulis* in an eelgrass meadow.. Journal of Experimental Marine Biology and Ecology.

[28] Dupont S, Thorndyke MC (2006). Growth or differentiation? Adaptive regeneration in the brittlestar *Amphiura filiformis*.. Journal of Experimental Biology.

[29] Verdu E, Ceballos D, Vilches JJ, Navarro X (2000). Influence of aging on peripheral nerve function and regeneration.. J Peripher Nerv Syst.

[30] Chen R, Cohen LG, Hallett M (2002). Nervous system reorganization following injury.. Neuroscience.

[31] Bollner T, Beesley PW, Thorndyke MC (1993). Distribution of GABA-like immunoreactivity during post-metamorphic development and regeneration of the central nervous system in the ascidian *Ciona intestinalis*.. Cell and Tissue Research.

[32] Chambost D (1966). Le complexe neural de *Ciona intestinalis*. Etude comparative du ganglion nerveux et de la glande asymétrique aux microscope optiques et électroniques.. C R Acad Sc Paris Série D.

[33] Koyama H, Kusunoki T (1993). Organization of the cerebral ganglion of the colonial ascidian *Polyandrocarpa misakiensis*.. Journal of Comparative Neurology.

[34] Akimenko MA, Mari-Beffa M, Becerra J, Geraudie J (2003). Old questions, new tools, and some answers to the mystery of fin regeneration.. Dev Dyn.

[35] Alvarado AS, Tsonis PA (2006). Bridging the regeneration gap: genetic insights from diverse animal models.. Nat Rev Genet.

[36] Tanaka EM (2003). Regeneration: if they can do it, why can't we?. Cell.

[37] Gould RM, Morrison HG, Gilland E, Campbell RK (2005). Myelin tetraspan family proteins but no non-tetraspan family proteins are present in the ascidian (*Ciona intestinalis*) genome.. Biol Bull.

[38] Ando R, Hama H, Yamamoto-Hino M, Mizuno H, Miyawaki A (2002). An optical marker based on the UV-induced green-to-red photoconversion of a fluorescent protein.. Proc Natl Acad Sci USA.

[39] Kumar A, Godwin JW, Gates PB, Garza-Garcia AA, Brockes JP (2007). Molecular basis for the nerve dependence of limb regeneration in an adult vertebrate.. Science.

