# Supplementary material for: Refining the Ciona intestinalis Model of Central Nervous System Regeneration
Source: PLoS One. 2009 Feb 12;4(2):e4458. doi: 10.1371/journal.pone.0004458 (PMC2639796; doi:10.1371/journal.pone.0004458)
Supplement: Table S1 — Preparation instructions (0.03 MB DOC) [file pone.0004458.s001.doc]

Table S1 Preparation instructions

| **Step** | **Action** |
| --- | --- |
| A | Anesthetize animal carefully and completely. |
| B | Remove tunic, be careful with regenerating animals. |
| C | Attach animal with thin needles (0.2 mm wolfram) through siphons and in each compartment. |
| D | Make an incision up the atrial siphon wall all the way through the opening. |
| E | Make an incision up the pharyngeal wall all the way through the opening. |
| F | Make sure needles fix the tissue. Hold right body wall with forceps and carefully cut the separator wall and pharynx wall until just before the ganglion (or hole). |
| G | Remove needles. |
| H | Move tissue preparation to a cover-glass bottomed chamber with the dorsal side towards the cover-glass. |
| I | Arrange tissues carefully remove possible extra tunic. |
| J | Carefully place a piece of slide glass on top of the tissue to weigh it down. |
| K | Fill chamber with water or media containing gentamycin and kanamycin. |
| L | Keep in correct temperature and change water twice daily. |

These preparations allow the user to do time lapse studies and can be kept alive for a long time.
